# Hypobaric type oxygenators during cardiopulmonary bypass for cardiac surgery to reduce gaseous microemboli: A randomized controlled trial

**DOI:** 10.1016/j.xjon.2026.101723

**Published:** 2026-03-12

**Authors:** Ben Gibbison, Phatiwat Chotimol, Helena Smartt, Barnaby C. Reeves, Alison Bamford, Barbara Warnes, Jessica Frost, Jodi Taylor, William Lansdowne, Gianni D. Angelini

**Affiliations:** a Bristol Medical School, University of Bristol, Bristol, United Kingdom; b Department of Anaesthesia, University Hospitals Bristol NHS Trust, Bristol, United Kingdom; c Bristol Trials Centre, University of Bristol, Bristol, United Kingdom; d Department of Clinical Perfusion, University Hospitals Bristol NHS Trust, Bristol, United Kingdom; e Bristol Heart Institute, University Hospitals Bristol NHS Trust, Bristol, United Kingdom

**Keywords:** cardiac surgery, cardiopulmonary bypass, oxygenator, stroke

## Abstract

**Objectives:**

Neurological complications after cardiac surgery have been associated with gaseous microemboli generated during cardiopulmonary bypass. We undertook a trial to assess whether a novel dual-chamber oxygenator, designed to denitrogenate the blood, could reduce cerebral gaseous microemboli compared with conventional cardiopulmonary bypass.

**Methods:**

In this single-center, single-blind, parallel-group randomized controlled trial, adults undergoing elective or urgent coronary artery bypass grafting with or without aortic valve replacement (±coronary artery bypass grafting) were assigned to the novel oxygenator or conventional cardiopulmonary bypass. Continuous intraoperative transcranial Doppler monitoring quantified cerebral gaseous microemboli in both middle cerebral arteries. Secondary outcomes included serum S100 Beta Protein Biomarker concentrations, cardiopulmonary bypass circuit gaseous microemboli counts, and 30-day adverse events.

**Results:**

The trial stopped early after recruiting 25 participants (coronary artery bypass grafting = 19; aortic valve replacement = 6) because elevated gaseous microemboli counts were not lower in the novel oxygenator group. Among patients undergoing coronary artery bypass grafting, median total gaseous microemboli counts were higher with the novel cardiopulmonary bypass than conventional cardiopulmonary bypass (right middle cerebral artery 170 [42-749] vs 129 [45-184]; left middle cerebral artery 234 [173-464] vs 93 [40-428]; incidence rate ratio 1.55; 95% CI, 0.64-3.74). No participant experienced neurological injury, and 1 patient died within 30 days.

**Conclusions:**

A novel, dual-chamber oxygenator for cardiopulmonary bypass did not reduce gaseous microemboli in the middle cerebral artery of patients during cardiac surgery.

**Figure:**
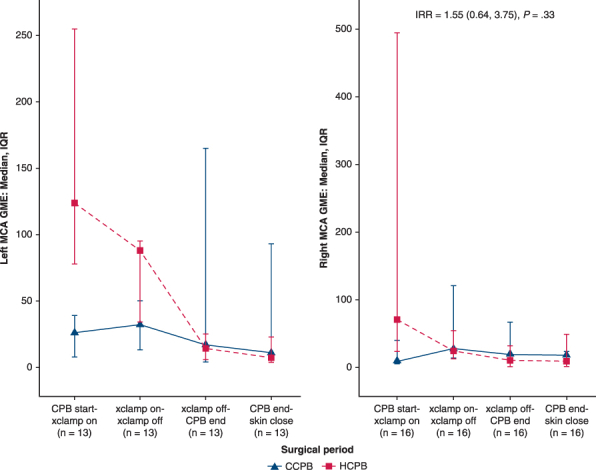
Number of GMEs detected by transcranial ultrasound in the MCAs using a conventional CPB oxygenator and a novel, dual-chamber HCPB.

**Central Message**

This early-phase trial found that a novel dual-chamber oxygenator designed to denitrogenate the blood did not reduce cerebral GME during CPB.

**Perspective**

GME remain a concern during CPB, with potential neurological consequences. In this early-phase randomized trial, a dual-chamber oxygenator designed to denitrogenate blood did not reduce microemboli counts, highlighting that promising technologies require careful evaluation and phased clinical testing before implementation.

Neurological complications are a major source of morbidity after cardiac surgery, with significant effects on both short- and long-term outcomes. Clinically overt stroke occurs in approximately 1% of patients,^1^ but magnetic resonance imaging evidence of cerebral ischemia is observed in up to 40%.^2^ Postoperative delirium is reported in approximately one-quarter of patients,^3^ and cognitive dysfunction at hospital discharge can be detected in approximately 40%.^4^ These neurological effects often extend into the long term, with approximately 25% of patients demonstrating measurable cognitive impairment at 6 months after surgery.^4^

A principal mechanism contributing to these complications is the formation and embolization of gaseous microemboli (GME) within the cardiopulmonary bypass (CPB) circuit. GMEs are small air bubbles, less than 200 μm in diameter, that are introduced into the circuit through several mechanisms. These include direct air entrainment during open-chamber surgery, cavitation within low-pressure, flow accelerating regions of the circuit (eg, at the roller/centrifugal pumps), and temperature gradients between the perfusate and the patient's core temperature. Nitrogen is the predominant component of GMEs, reflecting its lower solubility in plasma compared with oxygen and carbon dioxide.

One approach proposed to reduce GME formation involves the use of a hypobaric oxygenator.^5^ This system applies sub-atmospheric pressure to the oxygenator housing while delivering a sweep gas with a fraction of inspired oxygen of 1.0. The resulting partial pressure gradient enhances nitrogen diffusion from blood across the oxygenator membrane, promoting its clearance and potentially reducing GME generation. Adequate oxygenation is maintained by the high fraction of inspired oxygen, whereas the hypobaric pressure prevents over-oxygenation.

Spectrum Medical has developed a novel oxygenator designed to replicate the physiological principles of hypobaric oxygenation without the need for sub-atmospheric pressure.^6^ The Hypobaric type Oxygen Membrane Evaluation trial was undertaken to assess whether the Spectrum Quantum Oxygenator reduces the number of GMEs reaching the cerebral circulation and whether this reduction is associated with changes in cerebral and systemic inflammatory biomarkers and clinical outcomes.

## Material and Methods

The Hypobaric type Oxygen Membrane Evaluation trial was a single-center, single blind, parallel, 2-group, placebo-controlled randomized controlled trial. The setting was the Bristol Heart Institute at the University Hospitals Bristol and Weston NHS Foundation Trust in the United Kingdom. The protocol was reviewed by a National Health Service Research Ethics Committee and approved in April 2022 (IRAS ID 305038). The trial was registered as ISRCTN87042095 before starting to recruit participants.

### Participants

The trial population comprised adults undergoing elective or urgent isolated coronary artery bypass grafting (CABG) or aortic valve replacement (AVR) with or without an associated CABG procedure (AVR ± CABG) through a full sternotomy using central aortic perfusion cannulae for CPB. All participants were recruited after providing written informed consent for publication of study data. Inclusion criteria were age 40 years or more, and surgery was scheduled as an elective procedure. Exclusion criteria were previous cardiac surgery; medical history of clinical stroke within 3 months or myocardial infarction in the previous 4 days before surgery; cardiac catheterization within 1 day of the planned surgery; cerebral or aortic arch arteriography or interventions within 3 days of the planned surgery; active endocarditis at time of randomization; planned concomitant aortic procedure; clinical signs of cardiogenic shock or treatment with intravenous inotropic therapy before randomization; participation in an interventional (drug or device) trial; inability to provide written informed consent; and prisoners.

### Intervention and Comparator

Participants were randomly assigned to conventional CPB or the novel hypobaric type CPB. All CPB components used for both interventions are CE marked (a mandatory certification for products sold in the European Economic Area, signifying the manufacturer's declaration that the product meets European Union health, safety, and environmental standards). Conventional CPB comprised standard oxygenator, roller pump, hard-cell reservoir, arterial filter, shed-blood suctions, a range of venting options, uncoated tubing, and a cell-saver device. The following optional/alternative components could be integrated (and recorded accordingly): coated oxygenator, coated tubing and centrifugal pump. The following components were prohibited: soft-cell reservoir and vacuum-assisted venous drainage.

Hypobaric type CPB comprised the same circuit components as above but included a novel blood oxygenator device and inbuilt filter (Quantum Perfusion Dual Chamber Blood Oxygenator, Qura Srl) in place of the standard oxygenator. This oxygenator functions by having 2 sequential gas-exchange chambers both containing 100% oxygen. In the first (smaller) chamber, the available oxygen content is intentionally insufficient to fully saturate all hemoglobin on the blood side of the membrane such that hemoglobin saturations are below 98%. In the subsequent (larger) chamber, there is fine control of the hemoglobin saturation of oxygen to “top up” to 98% or greater. This 2-stage process enhances the concentration gradient for nitrogen diffusion from the blood (in that there is no nitrogen on the nonblood side of the membrane), thereby promoting denitrogenation from the blood. Software provided with the Dual-Chamber Oxygenator provides control of both arterial carbon dioxide and oxygen by automated adjustment of the sweep gas flow. There is a blood filter at the blood “exit” port of the oxygenator.

### Surgical and Cardiopulmonary Bypass Parameters

Nonpulsatile perfusion was maintained at a flow rate of 2.0 to 2.4 L min^−1^ m^−2^. Mean arterial pressure was maintained between 70 and 90 mm Hg, and patient temperature was maintained between 32 and 37 °C. Gas sweep flow was adjusted to maintain arterial carbon dioxide tension at 35 to 40 mm Hg (4.6-5.2 kPa) using the alpha-stat method. Carbon dioxide flooding of the cardiotomy field was not used. Standard cannulation involved arterial cannulation of the distal ascending aorta and insertion of a 2-stage venous cannula into the right atrium and inferior vena cava. After completion of the surgical procedure, the heart was passively de-aired through the aortic or left atrial suture line, and surgeons performed their routine de-airing maneuvers. All other aspects of the surgical technique were performed at the discretion of the operating surgeon and according to the operation type. The operating surgeon confirmed that these procedural elements were applied consistently across both the hypobaric type CPB and conventional CPB groups within each operative stratum.

### Outcomes

The primary outcome was the frequency of GME, measured continually during the surgery using transcranial Doppler ultrasound of the middle cerebral artery (MCA).^7^^,^^8^

Secondary outcomes were as follows:

1. Other transcranial Doppler data: particulate emboli, cluster showers, number of artefacts, cerebral blood flow velocity
2. Markers of inflammation and oxidative stress: tumor necrosis factor (TNF)-alpha, interleukin (IL)-6, IL-8, and IL-10 and the S100 Beta Protein Biomarker (S100β) proteins in serum
3. GME count in the CPB circuit (venous line, pre- and postoxygenator)
4. Serious adverse events to 30 days

Blood samples were obtained through existing intravenous lines preoperatively and at 1, 4, 12, and 24 hours postoperatively for biomarker measurements.

### Transcranial Doppler

All patients were fitted with a headframe before the day of surgery to ensure stable positioning of the transcranial Doppler (TCD) probes over the right and left temporal windows. Continuous intraoperative monitoring was performed using a dual-frequency TCD system (EmboDop, DWL) with 2.0 and 2.5 MHz probes targeting the middle cerebral arteries (MCAs) bilaterally. This multifrequency approach enabled automated detection and classification of microembolic signals as solid or gaseous using the device automated algorithm. This algorithm is based on differential backscatter (solid emboli exhibit greater reflectivity at higher frequencies, whereas gaseous emboli show the opposite), allowing accurate characterization of embolic composition. MCAs were found at depths of 30 to 65 mm, and cerebral blood flow velocity was recorded bilaterally. Monitoring started at the initiation of CPB and continued uninterrupted until skin closure, with data segmented into 4 predefined intraoperative periods: (1) start of CPB to aortic crossclamping; (2) crossclamping to clamp removal; (3) clamp removal to the end of CPB; and (4) end of CPB to skin closure. A subset of the TCD traces was reviewed by the manufacturers’ representative to ensure correct interpretation of solid versus GME and that diathermy artefact was correctly interpreted.

### Cardiopulmonary Bypass Circuit Bubble Count

Flow sensors were placed as standard on the venous line and pre- and postoxygenator. Bubbles were measured using ultrasound impedance by Spectrum Medical Flow-Sensors.^9^

### S100β, TNF-Alpha, IL-6, IL-8, and IL-10

Blood samples were collected preoperatively and at 1, 4, 12, and 24 hours postoperatively for analyses of biomarkers. Whole blood was drawn into a serum separator tube, clotted at room temperature for 30 minutes, and then immediately centrifuged at a relative centrifugal force of 2000*g* for 15 minutes. The supernatant containing serum was separated into a new 1.5 ml microcentrifuge tube and stored at −80 °C until assay after recruitment was complete. S100β levels in the serum were assayed using the Human S100β enzyme-linked immunosorbent assay method (Millipore) with a lower detection limit of the kit = 2.7 pg/mL. Samples were not assayed for TNF-alpha and ILs (see “Results” for full details).

### Data Collection

Data collection comprised the following elements:

1. A screening log of all patients undergoing cardiac surgery who fulfilled the inclusion criteria
2. Confirmation of participants' eligibility or, if ineligible, reasons for ineligibility
3. Baseline information (eg, medical history and assessments)
4. Details of the surgery and hospital stay
5. Details of any adverse event occurring up to 30 days after surgery

### Randomization and Blinding

Participants were randomized in a 1:1 ratio to hypobaric type CPB or conventional CPB stratified by type of surgery (CABG or AVR ± CABG). The randomization sequence was computer generated in advance using blocks of varying length by the trial statistician. Participants who consented were randomized as close to the planned operation as possible, after baseline assessments had been completed and eligibility confirmed by the clinical research team. Randomization was performed using a password-protected secure internet-based randomization system either the night before or on the day of surgery (at least 30 minutes before a participant was taken to the operating room). Information to identify a participant and to confirm eligibility had to be entered into the system before allocation was disclosed.

Participants were not informed of their allocations. Research staff collecting data, other than the clinical care team (perfusionist, anesthetist, surgeon, and TCDU operator), were blind to participants’ allocations. It was not possible to blind the clinical care team because the hypobaric type CPB and conventional CPB systems require different heater-cooler units.

### Statistical Analyses

The manufacturer of the hypobaric CPB device reports that it eliminates nitrogen from the arterial side of the CPB circuit and therefore should substantially reduce the number of GME entering the blood. In a previous randomized trial comparing conventional CPB with off-pump CABG, the geometric mean number of GME detected was 72 in the conventional CPB group and 4 in the off-pump group.^7^

Assuming that the number of GMEs during hypobaric type CPB would need to be similar to that observed during off-pump surgery to allow the hypobaric type CPB to be considered effective, we specified a large target difference. The SD on the natural log scale for the conventional CPB group in the previous study was 0.7 log units. A 10-fold reduction in transcranial Doppler ultrasound (TCDU) hits with hypobaric type CPB (equivalent to a difference of 2.3 log units) could be detected with 90% power using only 8 participants (4 in each group).

These calculations led us to choose a sample size to promote the generalizability of the results rather than to achieve 90% statistical power for a specified target difference. Therefore, we planned to include 20 participants undergoing CABG (10 hypobaric type CPB and 10 conventional CPB) and 20 participants undergoing AVR (AVR ± CABG; 10 hypobaric type CPB and 10 conventional CPB). Analyzing each procedure type as a parallel comparison (10 per group) provides 90% power to detect a 3-fold reduction in GME frequency.

All analyses followed a prespecified statistical analysis plan. The primary outcome and other TCD variables were summarized as mean (SD) or, where distributions were skewed, as median (interquartile range [IQR]). When the number of distinct count values was small, data were also summarized as frequencies and percentages of participants within each category.

TCD GME counts were compared between treatment groups using a negative binomial mixed-effects regression models, with treatment group and cerebral hemisphere (left/right MCA) included as fixed effects and participant as a random effect. Treatment effects are expressed as incidence rate ratios (IRRs) with 95% CIs. GME counts measured within the CPB circuit were summarized descriptively but not formally compared.

Binary outcomes were presented as numbers and percentages by treatment group. Postoperative serious adverse events were described descriptively and not compared formally because of the small number of events.

Serum S100β concentrations were summarized at each time point as mean (SD). Because data were positively skewed, values were log-transformed before analysis. Treatment effects were estimated using linear mixed-effects regression, including treatment group and preoperative concentrations as fixed effects and participant as a random effect. The time-by-treatment interaction met the prespecified threshold for inclusion (*P* < .10), and therefore separate treatment effect estimates with 95% CIs are reported for each time point. Results for log-transformed outcomes are presented as adjusted geometric mean ratios with 95% CIs. Protocol deviations, including nonadherence to randomized allocation, were described.

## Results

The study recruited patients between April 24, 2023, and November 9, 2023. Recruitment to the trial was suspended on November 9, 2023, because the unblinded TCDU operator identified a potential safety concern with the hypobaric type CPB circuit, that is, high numbers of both gaseous and particulate microemboli seen in the initial period of CPB until aortic crossclamping. The independent safety committee monitoring the trial reviewed the available data on December 6, 2023, and did not find actual harm to any participant. However, the committee identified that the available TCDU data demonstrated the futility of continuing the trial (see “Results” for the CABG group of participants below) and recommended that recruitment should not restart.

**Figure 1:**
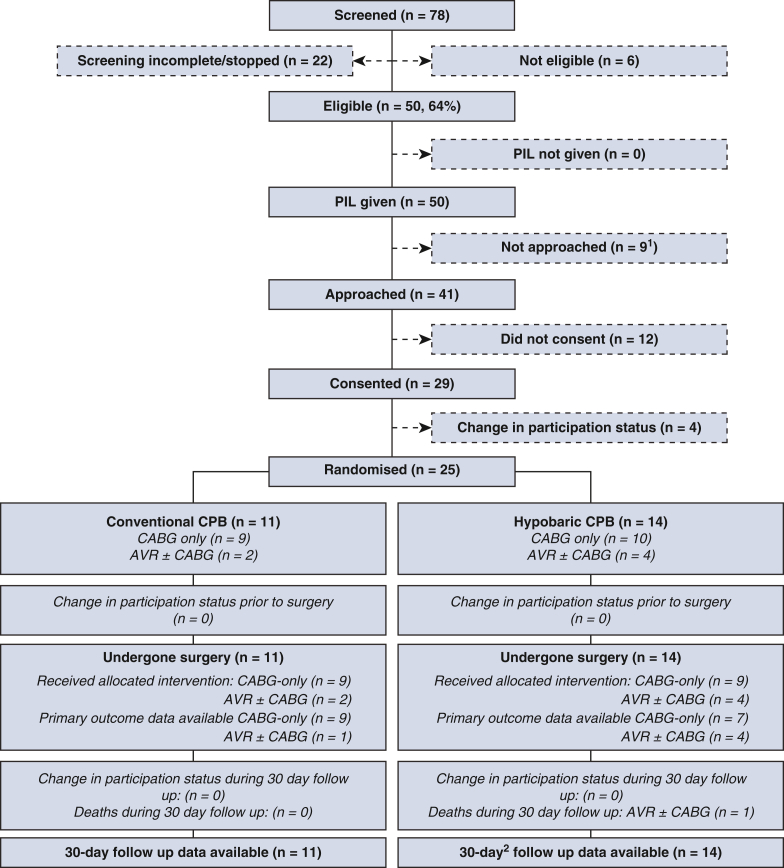
Patients’ flowchart. ^1^Approach status data obtained from file note for 1 patient. ^2^Follow-up data are available for the participant who died up to the time of death on day 23 postsurgery. *CPB*, Cardiopulmonary bypass; *CABG*, coronary artery bypass graft; *AVR*, aortic valve replacement.

When recruitment stopped, 25 participants had been randomized: 19 to the CABG stratum (9 conventional CPB, 10 to hypobaric type CPB) and 6 to the AVR stratum (2 conventional CPB and 4 hypobaric type CPB). Figure 1 shows screening and recruitment details.

Given the small number of participants undergoing AVR (n = 4), formal analyses of their data were not carried out. Their safety data are described below, and all other data for patients undergoing AVR are described in Tables E7-E12.

Baseline characteristics of CABG participants are shown in Table 1. Intraoperative details are shown in Table 2. Postoperative details are shown in Table 3. Protocol deviations and changes in participation status of consented participants before randomization are described in Tables E1 and E2.

**Table 1:** Baseline characteristics

Demography and quality of life	CABG only: Randomized to		Total (n = 19) n/N (%)
	CCPB (n = 9) n/N (%)	HCPB (n = 10) n/N (%)	
Baseline characteristics			
Female	1/9 (11%)	1/10 (10%)	2/19 (11%)
Age, y (mean, SD)	65.1 (7.3)	68.4 (8.7)	66.8 (8.0)
BMI (mean, SD)	27.8 (3.9)	26.7 (3.5)	27.2 (3.7)
Ethnicity			
White or Caucasian	9/9 (100%)	9/10 (90%)	18/19 (95%)
Other	0/9 (0%)	1/10 (10%)	1/19 (5%)
Medical history			
Smoking			
Yes	0/9 (0%)	1/10 (10%)	1/19 (5%)
Ex >1 mo	6/9 (67%)	5/10 (50%)	11/19 (58%)
No	3/9 (33%)	4/10 (40%)	7/19 (37%)
Diabetes			
Injected medication	0/9 (0%)	1/10 (10%)	1/19 (5%)
Oral	2/9 (22%)	5/10 (50%)	7/19 (37%)
No	7/9 (78%)	4/10 (40%)	11/19 (58%)
Hypothyroidism	0/9 (0%)	0/10 (0%)	0/19 (0%)
Cancer	2/9 (22%)	0/10 (0%)	2/19 (11%)
Chronic pulmonary disease	1/9 (11%)	2/10 (20%)	3/19 (16%)
Medically treated hypertension	7/9 (78%)	9/10 (90%)	16/19 (84%)
Medically treated hypercholesterolemia	5/9 (56%)	5/10 (50%)	10/19 (53%)
Family history (cardiovascular)	4/9 (44%)	2/10 (20%)	6/19 (32%)
Unstable angina	4/9 (44%)	2/10 (20%)	6/19 (32%)
Extracardiac arteriopathy	1/9 (11%)	1/10 (10%)	2/19 (11%)
Myocardial infarction	3/9 (33%)	5/10 (50%)	8/19 (42%)
Stroke	1/9 (11%)	0/10 (0%)	1/19 (5%)
Left ventricular ejection fraction			
Good (>50%)	7/9 (78%)	5/10 (50%)	12/19 (63%)
Moderate (30%-50%)	2/9 (22%)	5/10 (50%)	7/19 (37%)
>50% disease in the left main stem	2/9 (22%)	5/10 (50%)	7/19 (37%)
Heart rhythm			
Sinus	9/9 (100%)	10/10 (100%)	19/19 (100%)
Renal impairment			
Normal (CC >85 ml/min)	4/9 (44%)	7/10 (70%)	11/19 (58%)
Moderate (CC >85 and CC >50)	4/9 (44%)	3/10 (30%)	7/19 (37%)
Severe (CC <50)	1/9 (11%)	0/10 (0%)	1/19 (5%)
Poor mobility	0/9 (0%)	0/10 (0%)	0/19 (0%)
Previous cardiac surgery	0/9 (0%)	0/10 (0%)	0/19 (0%)
Active endocarditis	0/9 (0%)	0/10 (0%)	0/19 (0%)
Critical preoperative state	0/9 (0%)	0/10 (0%)	0/19 (0%)
NYHA class			
I	4/9 (44%)	2/10 (20%)	6/19 (32%)
II	3/9 (33%)	6/10 (60%)	9/19 (47%)
III	2/9 (22%)	1/10 (10%)	3/19 (16%)
IV	0/9 (0%)	1/10 (10%)	1/19 (5%)
CCS class			
I	1/9 (11%)	1/10 (10%)	2/19 (11%)
II	2/9 (22%)	7/10 (70%)	9/19 (47%)
III	5/9 (56%)	2/10 (20%)	7/19 (37%)
IV	1/9 (11%)	0/10 (0%)	1/19 (5%)
Pulmonary hypertension			
Moderate (PA systolic 31-55 mm Hg)	1/9 (11%)	0/10 (0%)	1/19 (5%)
None	8/9 (89%)	10/10 (100%)	18/19 (95%)
Operation-related factors			
Urgency			
Elective	1/9 (11%)	4/10 (40%)	5/19 (26%)
Urgent	8/9 (89%)	6/10 (60%)	14/19 (74%)
Weight of the intervention			
Isolated CABG	9/9 (100%)	10/10 (100%)	19/19 (100%)
Surgery of thoracic aorta	0/9 (0%)	0/10 (0%)	0/19 (0%)
euroSCORE II (%, median, IQR)	1.2 (0.9, 1.8)	1.2 (1.2, 1.7)	1.2 (0.9, 1.8)
Bloods			
Serum creatinine (μmol/L: median, IQR)	83 (77, 93)	85 (78, 88)	85 (77, 91)

*CABG*, Coronary artery bypass grafting; *CCPB*, conventional cardiopulmonary bypass; *HCPB*, hypobaric type cardiopulmonary bypass; *CC*, creatinine clearance; *NYHA*, New York Heart Association; *CCS*, Canadian Cardiovascular Society; *PA,* pulmonary artery; *euroSCORE*, European System for Cardiac Operative Risk Evaluation; *IQR*, interquartile range.

**Table 2:** Intraoperative details

Operation details	CABG only: Randomized to		Total (n = 19) n/N (%)
	CCPB (n = 9) n/N (%)	HCPB (n = 10) n/N (%)	
General operation details			
Duration of operation (h: mean, SD)	3.32 (0.2)	3.46 (0.6)	3.39 (0.5)
Total bypass time (min: mean, SD)	76 (15.9)	76 (15.5)	76 (15.3)
Total crossclamp time (min: mean, SD)	47 (12.4)	43 (11.7)	45 (11.8)
Type of surgical access			
Full sternotomy	9/9 (100.0%)	10/10 (100.0%)	19/19 (100.0%)
CABG: No. of anastomoses			
2	6/9 (66.7%)	4/10 (40.0%)	10/19 (52.6%)
3	3/9 (33.3%)	6/10 (60.0%)	9/19 (47.4%)
Lowest core body temperature recorded during operation (°C; mean, SD)	35.0 (1.4)	34.9 (0.7)	35.0 (1.1)
Insufflation details			
Carbon dioxide insufflation used	0/9 (0.0%)	0/10 (0.0%)	0/19 (0.0%)
Myocardial protection			
Myocardial protection type			
Blood	9/9 (100.0%)	8/10 (80.0%)	17/19 (89.5%)
Other	0/9 (0.0%)	2/10 (20.0%)	2/19 (10.5%)
Temperature			
Warm	2/9 (22.2%)	6/10 (60.0%)	8/19 (42.1%)
Cooled	7/9 (77.8%)	4/10 (40.0%)	11/19 (57.9%)
Infusion mode			
Antegrade	9/9 (100.0%)	10/10 (100.0%)	19/19 (100.0%)
Timing			
Intermittent	9/9 (100.0%)	10/10 (100.0%)	19/19 (100.0%)
Intraoperative complications			
Calcified aorta	0/9 (0.0%)	0/10 (0.0%)	0/19 (0.0%)
Postcardiotomy	0/9 (0.0%)	0/10 (0.0%)	0/19 (0.0%)

*CABG*, Coronary artery bypass grafting; *CCPB*, conventional cardiopulmonary bypass; *HCPB*, hypobaric type cardiopulmonary bypass.

**Table 3:** Postoperative details

Postoperative details	CABG only: Randomized to		Total (n = 19) n/N (%)
	CCPB (n = 9)	HCPB (n = 10)	
	n/N (%)	n/N (%)	
General postoperative details			
Postoperative intensive care stay length (h: median, IQR)	9.2 (6.8-12.9)	10.1 (6.6-11.4)	9.7 (6.6-12.9)
Postoperative hospital stay length (d: median, IQR)	6.1 (5.0-7.0)	6.6 (4.9-17.0)	6.1 (4.9-7.1)
Discharge details			
Discharge destination			
Hospital discharge home	9/9 (100.0%)	10/10 (100.0%)	19/19 (100.0%)
Discharge delayed	0/9 (0.0%)	0/10 (0.0%)	0/19 (0.0%)

*CABG*, Coronary artery bypass grafting; *CCPB*, conventional cardiopulmonary bypass; *HCPB*, hypobaric type cardiopulmonary bypass; *IQR*, interquartile range.

### Primary Outcome, Coronary Artery Bypass Grafting Group

**Figure 2:**
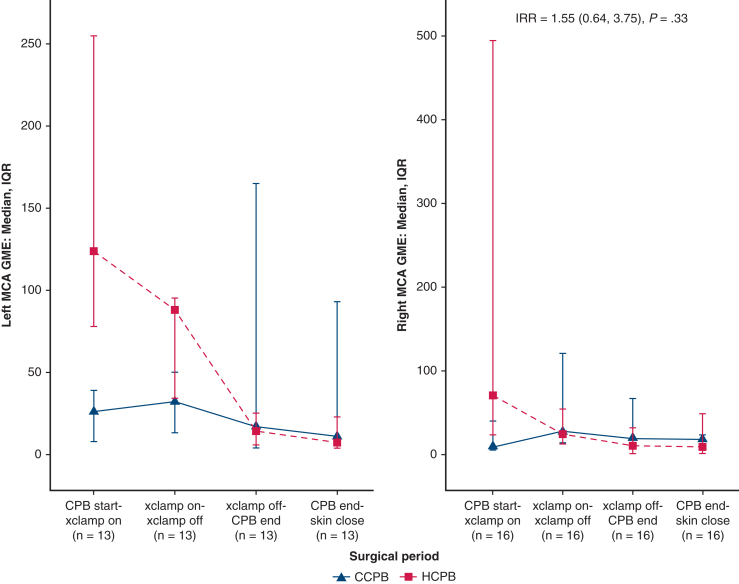
Number of GMEs detected by transcranial ultrasound in the MCAs using a conventional CPB oxygenator and a novel, dual-chamber hypobaric type CPB. *GME*, Gaseous microemboli; *MCA*, middle cerebral artery; *CPB*, cardiopulmonary bypass; *IQR*, interquartile range; *IRR*, incidence rate ratio.

The transcranial doppler GME observations for different phases of CABG surgery and overall are shown in Figure 2 and Table 4. The MCA could not be located in 3 patients (all 3 in the hypobaric type CPB group). Observation of the higher GME counts in patients undergoing CABG surgery in the hypobaric type CPB group, specifically from initiation of CPB to aortic crossclamping, was the trigger for the TCD operator raising concern (right MCA: conventional CPB median 9 [IQR, 6-40], hypobaric type CPB median 70 [IQR, 23-494], left MCA conventional CPB median 26 [IQR, 8-39], hypobaric type CPB median 124 [IQR, 78-255]). The formal analysis shows that the IRR of GME was higher in the hypobaric type CPB group but not to a statistically significant extent (IRR = 1.55, 95% CI, 0.64-3.74). The medians and IQRs show that the overall GME counts for participants in the hypobaric type CPB group were clearly higher than in the conventional CPB group. The same pattern was observed for solid microemboli (Table E3).

**Table 4:** Primary outcome: Transcranial Doppler ultrasound gaseous microemboli data

Outcome details	CABG only: Randomized to		Effect (95% CI)	*P* value
	CCPB (n = 9) Median, IQR	HCPB (n = 10) Median, IQR		
TCD scan completed (n/N (%))	9/9 (100.0%)	7/10 (70.0%)		
Reason TCD scan not completed (n/N (%))				
MCA could not be located		3/3 (100.0%)		
TCD counts available (n/N (%))				
Left MCA	7/9 (77.8%)	6/10 (60.0%)		
Right MCA	9/9 (100.0%)	7/10 (70.0%)		
Left MCA∗,†				
GME count from				
Start of CPB to crossclamp application	26 (8-39)	124 (78-255)		
Crossclamp application to crossclamp removal	32 (13-50)	88 (34-95)		
Crossclamp removal to discontinuation of CPB	17 (4-165)	14 (6-25)		
Discontinuation of CPB to skin closure	11 (5-93)	7 (4-23)		
Total GME count	93 (40-428)	234 (173-464)		
Total GME occurrence rate (counts/min)	0.8 (0.3-3.5)	1.7 (1.5-2.7)		
Right MCA∗				
GME count from				
Start of CPB to crossclamp application	9 (6-40)	70 (23-494)		
Crossclamp application to crossclamp removal	28 (14-121)	24 (13-54)		
Crossclamp removal to discontinuation of CPB	19 (11-67)	11 (1-32)		
Discontinuation of CPB to skin closure	18 (9-23)	9 (1-49)		
Total GME count	129 (45-184)	170 (42-749)		
Total GME occurrence rate (counts/min)	0.9 (0.4-1.3)	1.3 (0.4-4.5)		
Overall GME occurrence during surgery∗			IRR = 1.55 (0.64-3.75)	.33

*CABG*, Coronary artery bypass grafting; *CCPB*, conventional cardiopulmonary bypass; *HCPB*, hypobaric type cardiopulmonary bypass; *IQR*, interquartile range; *TCD*, transcranial Doppler; *MCA*, middle cerebral artery; *GME*, gaseous microemboli; *IRR*, incidence rate ratio.

∗ For all 4 time periods, data were missing for 3 participants where the MCA could not be located (3 HCPB).

† For all 4 time periods, data were missing for 3 participants (2 CCPB, 1 HCPB) for whom only the right MCA could be located.

### Secondary Outcomes, Coronary Artery Bypass Grafting Group

**Figure 3:**
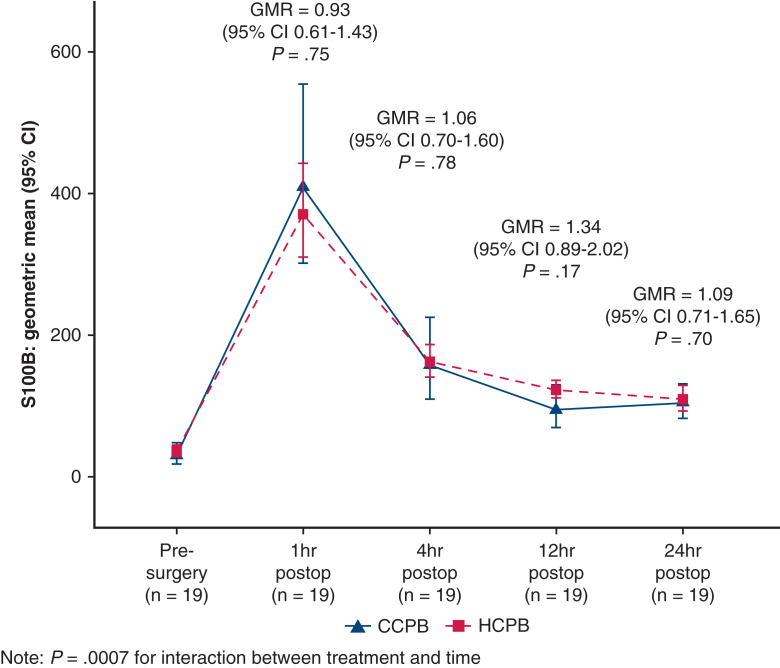
Serum S100β values at each measured time-point across the perioperative period. *P* = .0007 for interaction between treatment and time. *S100B*, S100 Beta Protein Biomarker; *CCPB*, conventional cardiopulmonary bypass; *HCPB*, hypobaric type cardiopulmonary bypass.

Serum S100ß levels at baseline, 1 hour, 4 hours, 12 hours, and 24 hours after surgery are shown in Figure 3. The interaction of intervention by time was statistically significant, and treatment estimates for each time point are reported (Figure 3 and Table E4). At all time points, there was no significant difference between groups; the interaction arose from the 12-hour time point when the mean level was nonsignificantly higher in the hypobaric type CPB group.

CPB circuit GME data are also reported in Table E4. The number of GME observed in the CPB circuit in both groups during CPB was low. However, there were more GMEs seen in the hypobaric type CPB group.

Blood samples were collected but not analyzed for anything other than S100β because their results would not have added meaningful value to the evaluation, given the observed GME counts in the hypobaric type CPB group. Serum samples for AVR participants were not assayed, given the small number of participants.

### Safety/Adverse Events

CABG participants in the hypobaric type CPB group experienced more in-hospital complications, but all participants in both groups experienced at least 1 complication. Three participants in each group experienced at least 1 serious complication. Summary details of the complications in the CABG group are shown in Table E4, with full details in Table E5. One patient in the AVR ± CABG strata (hypobaric type CPB) died of early endocarditis during the 30-day follow-up period. Full safety details of the AVR ± CABG group are shown in Tables E11 and E12. Details of follow-up and patients’ beliefs about treatment allocation group are shown in Table E6.

### Aortic Valve Replacement + Coronary Artery Bypass Group

Data for the 6 patients in this group are shown combined in Tables E7 to E12: baseline demography and baseline quality of life (Table E7); intraoperative and postoperative details (Table E8); TCD ultrasound GME data (Table E9); other TCD ultrasound data (Table E10); secondary outcome data (Table E11); and details of postoperative complications (Table E12).

## Discussion

The recruitment for this randomized study evaluating a novel CPB oxygenator and associated technology was terminated early after the observation of nonsignificantly higher GME counts in the MCA among participants assigned to the hypobaric type CPB group. Formal analysis of GME counts for CABG participants found a higher overall average count in the hypobaric type CPB group, but this was not statistically significantly different from the conventional CPB group. The high GME counts in hypobaric type CPB participants were most pronounced during the initial phase of CPB (from the start of bypass to aortic crossclamping). Increased GME counts were also detected within the CPB circuit itself when the novel hypobaric type oxygenator was used. Serum S100β concentrations and other clinical outcomes did not differ substantially between the study groups.

Although no participant experienced any obvious clinical harm, recruitment was not resumed on precautionary grounds. The monitoring committee determined that further enrollment would be futile, because the existing data were sufficient to demonstrate that the novel oxygenator was not effective in reducing GME to a lower level at any point during CPB. To our knowledge, there are no published clinical studies have evaluated the Quantum Oxygenator or similar devices, precluding direct comparison with previous human data. Experimental studies of true hypobaric oxygenators in animal models have shown reductions in blood nitrogen partial pressures and subsequent decreases in cerebral GME formation.^5^^,^^10^^,^^11^ However, such findings have not been validated in humans.

One potential source of GME during the early CPB period is residual air remaining in the CPB circuit or oxygenator. We adhered to the manufacturer's published priming protocol during the study, which was supervised by the manufacturer's representative during initial use. If residual air were the primary cause, we would expect the GME counts to decline substantially in the later phases of CPB to levels lower than those seen with conventional CPB, but this reduction did not occur.

It is worth noting that new CPB equipment is subject to Medicines and Healthcare Products Regulatory Agency approval as a medical device before market release (Class IIb). These devices (eg, oxygenators, tubing and filters) require conformity testing for commercial distribution and may not have undergone formal testing in patients, a regulatory approach that also applies to a range of other devices.

Following our findings, we engaged in discussion with the manufacturer, who conducted further testing of the oxygenator and did not replicate the early GME generation observed in patients. After reevaluating our CPB circuit setup (including priming procedure) and obtaining external review of the TCD recordings (both of which were confirmed as correct), we concluded that the observed findings were genuine.

The study was a randomized controlled trial with concealed randomization, reducing bias from assignment of participants to groups. The trial was conducted according to a protocol, and statistical analyses adhered to a prespecified statistical plan, minimizing reporting bias. TCD-based GME data (the primary outcome) were externally validated by the device suppliers, and serum S100 B levels were assayed “blind.” All participants but 1 were followed to the scheduled end of follow-up.

### Limitations

This study has some limitations. First, it was a single center with a small sample size. That said, the nonsignificant differences during early CPB (and lack of difference at later time-points) in GME counts suggest there is little if any benefit to using the new oxygenator. Second, we were unable to fully blind the outcome assessor, and there may be a risk of operator bias. However, the GME counts by the transcranial Doppler device are detected and recorded via an electronic algorithm within the device and are not subject to manipulation by the operator. The operator fits the device and removes artefact. There were differences between the amount of artefact in the hypobaric and conventional CPB groups (higher in the hypobaric type CPB group). A subset of traces were inspected and independently confirmed as correct by the supplier of the TCD device, suggesting that these apparent differences are genuine. Although there was a difference in artefact, the direction of the effect would tend to reduce the observed GME count in the hypobaric type CPB group. This enhances our findings, in that artefact would have obscured more GME, and therefore we are likely to have undercounted the number of GME in the hypobaric type CPB group.

There were a small number of protocol deviations, including 1 participant who did not receive the allocated intervention. These deviations are unlikely to have materially influenced the study findings. Primary outcome data were available for 7 participants in the hypobaric group. However, given the magnitude and consistency of the observed effect, this limitation is also unlikely to have materially affected the overall conclusions.

The implications of this study are that the Quantum Oxygenator should undergo further evaluation before widespread clinical use to optimize elimination of GME. Although true hypobaric oxygenators are an attractive solution to reduction of GME and potential neurological injury after cardiac surgery, they need to undergo first-in-human testing before widespread clinical use.

## Conclusions

In this single-center, randomized controlled trial, a novel, dual-chamber oxygenator for CPB did not reduce GME in the MCA of patients. Further evaluation is required to determine the cause and mechanism of these findings.

## Conflict of Interest Statement

The authors reported no conflicts of interest.

The *Journal* policy requires editors and reviewers to disclose conflicts of interest and to decline handling or reviewing manuscripts for which they may have a conflict of interest. The editors and reviewers of this article have no conflicts of interest.
