# Supplementary material for: Hypobaric type oxygenators during cardiopulmonary bypass for cardiac surgery to reduce gaseous microemboli: A randomized controlled trial
Source: JTCVS Open. 2026 Mar 12;31:101723. doi: 10.1016/j.xjon.2026.101723 (PMC13316339; doi:10.1016/j.xjon.2026.101723)
Supplement: Online Data Supplement [file mmc1.docx]

**Supplementary file**

*De-airing manoeuvres:*

With the left ventricle (LV) and vent clamped, the heart was filled with blood by occluding the venous return, and the lungs were ventilated while maintaining suction on the aortic root vent. Complimentary de-airing of the apex was achieved by brief and low suction on the LV vent, which was stopped before removal of the aortic clamp. After removal of the cross clamp, the aortic suction vent is maintained at 200-300ml/min. The LV vent could be opened for brief periods if the LV distended, but prolonged suction was avoided when the heart is empty to prevent air from entering the LA around the vent. Gravity drainage of the LV vent (rather than suction) prevented air from being entrained and is acceptable when the heart is empty. Additional deairing during the rewarming period was achieved by maintaining aortic root vent suction.

When the transoesophageal echo showed that all microbubbles had been evacuated, the LV vent was removed. The aortic root vent was maintained until the patient was totally weaned from cardiopulmonary bypass.

All other aspects of the participant’s pre, intra and post-operative management were in accordance with existing protocols.

**Table S1: Protocol deviations in the CABG only group**

|  | **CABG only: randomised to:-** | | **Total** |
| --- | --- | --- | --- |
|  | **CCPB (n=9)** | **HCPB (n=10)** | **(n=19)** |
| **Deviation description** | **n/N (%)** | **n/N (%)** | **n/N (%)** |
| Allocated intervention not received | 0/9 (0%) | 1/10 (10%) | 1/19 (5%) |
| Anaesthesia or Myocardial protection  procedures specified in protocol not  used | 3/9 (33%) | 3/10 (30%) | 6/19 (32%) |
| Manual randomisation | 0/9 (0%) | 1/10 (10%) | 1/19 (5%) |
| Less than 12 hours available to consider taking part in study | 2/9 (22%) | 0/10 (0%) | 2/19 (11%) |

*Note: CABG: Coronary Artery Bypass Graft, CCPB: conventional cardiopulmonary bypass, HCPB: hypobaric cardiopulmonary bypass*

**Table S2: Changes in participation status prior to randomisation in the CABG only group**

| **Participant number** | **Person(s) responsible for change in participation status:-** | **Reason for change in participation status** |
| --- | --- | --- |
| 1 | Study team | Operation carried out by non-study surgeon |
| 2 | Study team | No perfusionist available on day of surgery |
| 3 | Study team | Operation performed at private hospital |
| 4 | Study team | Operation performed on a weekend |

*Note: no changes in participation status took place after randomisation for any participants*

**Table S3: Other Trans-cranial doppler ultrasound (TCDU) data for the CABG only group**

|  | **CABG only: randomised to:-** | | **Total** |
| --- | --- | --- | --- |
|  | **CCPB (n=9)** | **HCPB (n=10)** | **(n=19)** |
| **Outcome details** | **Median, IQR** | **Median, IQR** | **Median, IQR** |
| **PARTICULATE MICROEMBOLI (PME):-** | |  |  |
| **Right middle cerebral artery**^1^  PME count from:- |  |  |  |
| start of CPB to cross-clamp application | 4 (3, 6) | 62 (13, 187) | 6 (4, 48) |
| cross-clamp application to cross-clamp removal | 3 (2, 10) | 5 (4, 63) | 4 (2, 13) |
| cross-clamp removal to discontinuation of CPB | 2 (1, 6) | 3 (1, 17) | 2 (1, 12) |
| discontinuation of CPB to skin closure | 6 (1, 9) | 5 (0, 29) | 6 (0, 9) |
| Total PME count | 18 (15, 20) | 171 (33, 209) | 26 (15, 133) |
| Total PME occurrence rate (counts/min) | 0.1 (0.1, 0.2) | 1.2 (0.4, 1.6) | 0.3 (0.1, 1.0) |
| **Left middle cerebral artery**^2^  GME count from:- |  |  |  |
| start of CPB to cross-clamp application | 7 (1, 11) | 92 (49, 126) | 13 (7, 70) |
| cross-clamp application to cross-clamp removal | 5 (3, 10) | 58 (23, 62) | 10 (5, 55) |
| cross-clamp removal to discontinuation of CPB | 9 (0, 36) | 10 (4, 19) | 9 (4, 19) |
| discontinuation of CPB to skin closure | 2 (0, 15) | 2 (1, 4) | 2 (1, 5) |
| Total GME count | 31 (15, 54) | 164 (116, 208) | 59 (31, 131) |
| Total GME occurrence rate (counts/min) | 0.3 (0.1, 0.4) | 1.2 (1.0, 1.5) | 0.6 (0.3, 1.0) |
| **ARTEFACTS** | |  |  |
| **Right middle cerebral artery**^1^  Artefact count from:- |  |  |  |
| start of CPB to cross-clamp application | 0 (0, 1) | 0 (0, 201) | 0 (0, 4) |
| cross-clamp application to cross-clamp removal | 0 (0, 0) | 2 (0, 68) | 0 (0, 1) |
| cross-clamp removal to discontinuation of CPB | 0 (0, 0) | 0 (0, 7) | 0 (0, 0) |
| discontinuation of CPB to skin closure | 5 (0, 45) | 71 (1, 141) | 21 (1, 95) |
| Total Artefact count | 5 (2, 51) | 73 (5, 472) | 42 (4, 165) |
| Total Artefact occurrence rate (counts/min) | 0.0 (0.0, 0.4) | 0.4 (0.0, 5.1) | 0.3 (0.0, 1.3) |
| **Left middle cerebral artery**^2^  Artefact count from:- |  |  |  |
| start of CPB to cross-clamp application | 0 (0, 3) | 0 (0, 202) | 0 (0, 3) |
| cross-clamp application to cross-clamp removal | 0 (0, 0) | 1 (0, 68) | 0 (0, 1) |
| cross-clamp removal to discontinuation of CPB | 0 (0, 5) | 0 (0, 5) | 0 (0, 5) |
| discontinuation of CPB to skin closure | 9 (0, 36) | 110 (29, 140) | 29 (1, 126) |
| Total Artefact count | 23 (0, 204) | 114 (29, 472) | 36 (10, 204) |
| Total Artefact occurrence rate (counts/min) | 0.2 (0.0, 1.4) | 0.9 (0.2, 5.1) | 0.3 (0.1, 1.4) |
| **CLUSTER SHOWER** | |  |  |
| **Right middle cerebral artery**^1^  Cluster shower duration (sec) from:- |  |  |  |
| start of CPB to cross-clamp application | 2 (0, 8) | 9 (2, 55) | 3 (0, 14) |
| cross-clamp application to cross-clamp removal | 0 (0, 0) | 0 (0, 4) | 0 (0, 0) |
| cross-clamp removal to discontinuation of CPB | 0 (0, 17) | 0 (0, 0) | 0 (0, 8) |
| discontinuation of CPB to skin closure | 0 (0, 0) | 0 (0, 0) | 0 (0, 0) |
| **Left middle cerebral artery**^2^  Cluster shower duration (sec) from:- |  |  |  |
| start of CPB to cross-clamp application | 0 (0, 10) | 72 (55, 82) | 10 (0, 66) |
| cross-clamp application to cross-clamp removal | 0 (0, 0) | 13 (0, 34) | 0 (0, 3) |
| cross-clamp removal to discontinuation of CPB | 0 (0, 27) | 0 (0, 0) | 0 (0, 14) |
| discontinuation of CPB to skin closure | 0 (0, 0) | 0 (0, 0) | 0 (0, 0) |
| **TIME-AVERAGED MAXIMUM FLOW VELOCITY** | |  |  |
| **Right middle cerebral artery**^1^  Time-averaged maximum flow velocity (L/min) from:- |  |  |  |
| start of CPB to cross-clamp application | 35 (31, 45) | 24 (19, 46) | 32 (22, 46) |
| cross-clamp application to cross-clamp removal | 36 (34, 40) | 27 (16, 36) | 34 (26, 38) |
| cross-clamp removal to discontinuation of CPB | 36 (31, 42) | 25 (17, 31) | 31 (22, 36) |
| discontinuation of CPB to skin closure | 46 (40, 51) | 38 (29, 40) | 40 (30, 50) |
| **Left middle cerebral artery**^2^  Time-averaged maximum flow velocity (L/min) from:- |  |  |  |
| start of CPB to cross-clamp application | 36 (27, 46) | 36 (31, 54) | 36 (29, 46) |
| cross-clamp application to cross-clamp removal | 37 (32, 39) | 38 (28, 39) | 37 (32, 39) |
| cross-clamp removal to discontinuation of CPB | 42 (28, 48) | 34 (28, 39) | 35 (28, 44) |
| discontinuation of CPB to skin closure | 41 (37, 50) | 44 (35, 53) | 41 (37, 50) |

*Note: CABG: Coronary Artery Bypass Graft, CCPB: conventional cardiopulmonary bypass, HCPB: hypobaric cardiopulmonary bypass, IQR: Interquartile Range, GME: Gaseous Micro-emboli, PME: Particulate Micro-emboli.*

*^1^ For all four time periods, data were missing for 3 participants where the middle cerebral artery could not be located (3 HCPB)*

*^2^ For all four time periods, data were missing for 3 participants where the middle cerebral artery could not be located (3 HCPB) and three further participants (2 CCPB, 1 HCPB) for whom only the right middle cerebral artery could be located*

**Table S4: Secondary outcome data for the CABG only group.**

|  | **CABG only: randomised to:-** | |  |  |
| --- | --- | --- | --- | --- |
|  | **CCPB (n=9)** | **HCPB (n=10)** | **Effect** | **p-** |
| **Outcome details** | **n/N (%)** | **n/N (%)** | **(95% CI)** | **Value** |
| **Cardiopulmonary bypass circuit (CPB) gaseous microemboli (GME)** | | |  |  |
| **Number of CPB circuit GME (from**  **CPB start to 10 minutes after CPB**  **end)**^1^**:** |  |  |  |  |
| Size 1 |  |  |  |  |
| *0* | 5/8 (62%) | 3/10 (30%) |  |  |
| *1* | 2/8 (25%) | 3/10 (30%) |  |  |
| *2* | 1/8 (12%) | 2/10 (20%) |  |  |
| *4* | 0/8 (0%) | 1/10 (10%) |  |  |
| *8* | 0/8 (0%) | 1/10 (10%) |  |  |
| Size 2 |  |  |  |  |
| *0* | 7/8 (88%) | 5/10 (50%) |  |  |
| *1* | 1/8 (12%) | 3/10 (30%) |  |  |
| *2* | 0/8 (0%) | 1/10 (10%) |  |  |
| *3* | 0/8 (0%) | 1/10 (10%) |  |  |
| Size 3 |  |  |  |  |
| *0* | 7/8 (88%) | 7/10 (70%) |  |  |
| *1* | 1/8 (12%) | 1/10 (10%) |  |  |
| *2* | 0/8 (0%) | 2/10 (20%) |  |  |
| All sizes |  |  |  |  |
| *0* | 5/8 (62%) | 1/10 (10%) |  |  |
| *1* | 2/8 (25%) | 4/10 (40%) |  |  |
| *2* | 0/8 (0%) | 1/10 (10%) |  |  |
| *4* | 1/8 (12%) | 2/10 (20%) |  |  |
| *5* | 0/8 (0%) | 1/10 (10%) |  |  |
| *13* | 0/8 (0%) | 1/10 (10%) |  |  |
| **Rate of CPB circuit GME occurrence**  **(from CPB start to 10 minutes after**  **CPB end, counts/hour: median,IQR)**^1^ |  |  |  |  |
| *Size 1* | 0.0 (0.0, 1.0) | 1.2 (0.0, 1.3) |  |  |
| *Size 2* | 0.0 (0.0, 0.0) | 0.3 (0.0, 0.8) |  |  |
| *Size 3* | 0.0 (0.0, 0.0) | 0.0 (0.0, 0.7) |  |  |
| *All sizes* | 0.0 (0.0, 1.0) | 1.3 (0.8, 3.1) |  |  |
| **Serum S100B levels (GM, 95% CI; pg/ml)** | | |  |  |
| Pre-surgery | 30 (15,59) | 36 (27,49) |  |  |
| 1h post-operative | 408 (262,636) | 371 (287,479) | GMR=0.93, (0.61, 1.43) | 0.75 |
| 4h post-operative | 158 (93,266) | 162 (133,199) | GMR=1.06, (0.70, 1.60) | 0.78 |
| 12h post-operative | 95 (61,148) | 124 (108,141) | GMR=1.34, (0.89, 2.02) | 0.17 |
| 24h post-operative | 104 (76,144) | 110 (86,140) | GMR=1.09, (0.71, 1.65) | 0.70 |
| Test for treatment*time interaction |  |  |  | 0.0007 |
| **Adverse events within 30 days of surgery** | | |  |  |
| Number of in-hospital complications  (events/participants) | 30/9 (100%) | 66/10 (100%) |  |  |
| Number of serious^2^ in-hospital  complications (events/participants) | 3/3 (33%) | 11/3 (30%) |  |  |
| Number of hospital readmissions  within 30 days of surgery | 2/9 (22%) | 1/10 (10%) |  |  |
| Number of SAEs^2^ experienced  during 30-day follow-up period  (events/patients) | 9/4 (44%) | 12/3 (30%) |  |  |

*Note: CABG: Coronary Artery Bypass Graft, CCPB: conventional cardiopulmonary bypass, HCPB: hypobaric cardiopulmonary bypass, GME: Gaseous Micro-emboli, IQR: Interquartile Range, GM: Geometric mean, CI: Confidence Interval, GMR: Geometric Mean Ratio, SAE: Serious adverse event.*

*^1^ CPB data were not available for one participant (CCPB)*

*^2^ serious adverse events are defined as adverse events leading to or prolonging an existing hospitalisation, resulting in persistent or significant disability, leading to congenital anomaly/birth defect, that are life-threatening or cause death, or that constitute another significant medical event*

**Table S5: Detail of postoperative complications for the CABG only group (by treatment received)**

|  | **CABG only** | | | |  | |
| --- | --- | --- | --- | --- | --- | --- |
|  | **CCPB received**  **(n=9)** | | **HCPB received^1^**  **(n=9)** | | **Overall (n=18)** | |
| **Complication type (by MedDRA preferred term)** | **Complications**  **Events/ Participants (%)** | **Serious^2^ complications**  **Events/ Participants (%)** | **Complications**  **Events/ Participants (%)** | **Serious complications**  **Events/ Participants (%)** | **Complications**  **Events/ Participants (%)** | **Serious complications**  **Events/ Participants (%)** |
| **IN HOSPITAL COMPLICATIONS** |  |  |  |  |  |  |
| *Cardiac disorders* |  |  |  |  |  |  |
| Arrhythmia | 3/3 (33%) | 0/0 (0%) | 9/7 (78%) | 3/1 (11%) | 12/10 (56%) | 3/1 (6%) |
| Cardiac failure congestive | 0/0 (0%) | 0/0 (0%) | 1/1 (11%) | 1/1 (11%) | 1/1 (6%) | 1/1 (6%) |
| Pericardial effusion | 0/0 (0%) | 0/0 (0%) | 1/1 (11%) | 0/0 (0%) | 1/1 (6%) | 0/0 (0%) |
| *Gastrointestinal disorders* |  |  |  |  |  |  |
| Constipation | 2/2 (22%) | 0/0 (0%) | 0/0 (0%) | 0/0 (0%) | 2/2 (11%) | 0/0 (0%) |
| Diarrhoea | 1/1 (11%) | 0/0 (0%) | 0/0 (0%) | 0/0 (0%) | 1/1 (6%) | 0/0 (0%) |
| Nausea | 0/0 (0%) | 0/0 (0%) | 3/3 (33%) | 0/0 (0%) | 3/3 (17%) | 0/0 (0%) |
| Vomiting | 0/0 (0%) | 0/0 (0%) | 2/2 (22%) | 0/0 (0%) | 2/2 (11%) | 0/0 (0%) |
| *General disorders and administration site conditions* | | |  |  |  |  |
| Chest pain | 2/2 (22%) | 0/0 (0%) | 0/0 (0%) | 0/0 (0%) | 2/2 (11%) | 0/0 (0%) |
| Peripheral oedema | 2/2 (22%) | 0/0 (0%) | 7/7 (78%) | 0/0 (0%) | 9/9 (50%) | 0/0 (0%) |
| Post procedural fever | 1/1 (11%) | 0/0 (0%) | 2/2 (22%) | 0/0 (0%) | 3/3 (17%) | 0/0 (0%) |
| *Infections and infestations* |  |  |  |  |  |  |
| Infection | 0/0 (0%) | 0/0 (0%) | 1/1 (11%) | 1/1 (11%) | 1/1 (6%) | 1/1 (6%) |
| Pneumonia | 1/1 (11%) | 0/0 (0%) | 1/1 (11%) | 0/0 (0%) | 2/2 (11%) | 0/0 (0%) |
| *Injury, poisoning and procedural complications* | |  |  |  |  |  |
| Fall | 0/0 (0%) | 0/0 (0%) | 1/1 (11%) | 0/0 (0%) | 1/1 (6%) | 0/0 (0%) |
| Post procedural haemorrhage | 0/0 (0%) | 0/0 (0%) | 1/1 (11%) | 0/0 (0%) | 1/1 (6%) | 0/0 (0%) |
| Surgical procedure repeated | 0/0 (0%) | 0/0 (0%) | 1/1 (11%) | 1/1 (11%) | 1/1 (6%) | 1/1 (6%) |
| *Metabolism and nutrition disorders* | |  |  |  |  |  |
| Hypoglycaemia | 0/0 (0%) | 0/0 (0%) | 1/1 (11%) | 0/0 (0%) | 1/1 (6%) | 0/0 (0%) |
| Hyperglycaemia | 0/0 (0%) | 0/0 (0%) | 1/1 (11%) | 0/0 (0%) | 1/1 (6%) | 0/0 (0%) |
| Hyponatraemia | 1/1 (11%) | 0/0 (0%) | 1/1 (11%) | 0/0 (0%) | 2/2 (11%) | 0/0 (0%) |
| *Psychiatric disorders* |  |  |  |  |  |  |
| Hallucination | 1/1 (11%) | 1/1 (11%) | 0/0 (0%) | 0/0 (0%) | 1/1 (6%) | 1/1 (6%) |
| *Renal and urinary disorders* |  |  |  |  |  |  |
| Acute kidney injury | 1/1 (11%) | 1/1 (11%) | 1/1 (11%) | 0/0 (0%) | 2/2 (11%) | 1/1 (6%) |
| *Respiratory, thoracic and mediastinal disorders* | |  |  |  |  |  |
| Atelectasis | 2/2 (22%) | 0/0 (0%) | 1/1 (11%) | 0/0 (0%) | 3/3 (17%) | 0/0 (0%) |
| Pleural effusion | 4/4 (44%) | 1/1 (11%) | 2/2 (22%) | 0/0 (0%) | 6/6 (33%) | 1/1 (6%) |
| Pneumothorax | 0/0 (0%) | 0/0 (0%) | 3/2 (22%) | 1/1 (11%) | 3/2 (11%) | 1/1 (6%) |
| *Surgical and medical procedures* |  |  |  |  |  |  |
| Oxygen therapy | 0/0 (0%) | 0/0 (0%) | 2/2 (22%) | 0/0 (0%) | 2/2 (11%) | 0/0 (0%) |
| *Vascular disorders* |  |  |  |  |  |  |
| Haemodynamic instability | 8/8 (89%) | 0/0 (0%) | 9/9 (100%) | 0/0 (0%) | 17/17 (94%) | 0/0 (0%) |
| Hypertension | 1/1 (11%) | 0/0 (0%) | 0/0 (0%) | 0/0 (0%) | 1/1 (6%) | 0/0 (0%) |
| Venous thrombosis | 0/0 (0%) | 0/0 (0%) | 1/1 (11%) | 0/0 (0%) | 1/1 (6%) | 0/0 (0%) |
| *TOTAL IN-HOSPITAL COMPLICATIONS* | 30/9 (100%) | 3/3 (33%) | 52/9 (100%) | 7/2 (22%) | 82/18 (100%) | 10/5 (28%) |
| **ADVERSE EVENTS FROM DISCHARGE TO 30 DAYS AFTER SURGERY** | | |  |  |  |  |
| *Gastrointestinal disorders* |  |  |  |  |  |  |
| Diarrhoea | 1/1 (11%) | 1/1 (11%) | 0/0 (0%) | 0/0 (0%) | 1/1 (6%) | 1/1 (6%) |
| *Infections and infestations* |  |  |  |  |  |  |
| Infection | 1/1 (11%) | 1/1 (11%) | 0/0 (0%) | 0/0 (0%) | 1/1 (6%) | 1/1 (6%) |
| *Renal and urinary disorders* |  |  |  |  |  |  |
| Acute kidney injury | 1/1 (11%) | 1/1 (11%) | 0/0 (0%) | 0/0 (0%) | 1/1 (6%) | 1/1 (6%) |
| *Respiratory, thoracic and mediastinal disorders* | |  |  |  |  |  |
| Pleural effusion | 1/1 (11%) | 1/1 (11%) | 0/0 (0%) | 0/0 (0%) | 1/1 (6%) | 1/1 (6%) |
| *Surgical and medical procedures* |  |  |  |  |  |  |
| Haemofiltration | 1/1 (11%) | 1/1 (11%) | 0/0 (0%) | 0/0 (0%) | 1/1 (6%) | 1/1 (6%) |
| *Vascular disorders* |  |  |  |  |  |  |
| Hypotension | 1/1 (11%) | 1/1 (11%) | 0/0 (0%) | 0/0 (0%) | 1/1 (6%) | 1/1 (6%) |
| *TOTAL POST-DISCHARGE COMPLICATIONS* | 6/2 (100%) | 6/2 (100%) | 0/0 (0%) | 0/0 (0%) | 6/2 (100%) | 6/2 (100%) |
| ***OVERALL (TOTAL COMPLICATIONS DURING 30 DAY FOLLOW-UP)*** | 36/9 (100.0%) | 9/4 (44.4%) | 52/9 (100.0%) | 7/2 (22.2%) | 88/18 (100.0%) | 16/6 (33.3%) |

*Note: CABG: Coronary Artery Bypass Graft, CCPB: conventional cardiopulmonary bypass, HCPB: hypobaric cardiopulmonary bypass, SAE: Serious adverse event,MedDRA: Medical Dictionary for Regulatory Activities*

*^1^ One participant allocated to HCPB did not receive the allocated intervention (and is not included in the table above). They experienced non-serious adverse events of arrhythmia, peripheral oedema, wound dehiscence, bronchoscopy, somnolence, agitation, delirium, atelectasis, pleural effusion and haemodynamic instability and serious adverse events of cardiac arrest, pneumonia, a procedural complication and a repeated surgical procedure during their index hospital admission. After discharge they also experienced a serious complication of a wound infection.*

*^2^ serious adverse events are defined as adverse events leading to or prolonging an existing hospitalisation, resulting in persistent or significant disability, leading to congenital anomaly/birth defect, that are life-threatening or cause death, or that constitute another significant medical event*

**Table S6: Follow-up data for the CABG only group.**

|  | **CABG only** | | **Total** |
| --- | --- | --- | --- |
|  | **CCPB (n=9)** | **HCPB (n=10)** | **(n=19)** |
| **Follow-up details** | **n/N (%)** | **n/N (%)** | **n/N (%)** |
| **General** |  |  |  |
| Follow up call completed | 9/9 (100.0%) | 9/10 (90.0%) | 18/19 (94.7%) |
| Reason follow up call not completed |  |  |  |
| *Unable to contact^1^* | - | 1/1 (100.0%) | 1/1 (100.0%) |
| Readmitted to hospital? | 2/9 (22.2%) | 1/10 (10.0%) | 3/19 (15.8%) |
| Length of hospital readmission (days) |  |  |  |
| *6* | 0/2 (0.0%) | 1/1 (100.0%) | 1/3 (33.3%) |
| *9* | 1/2 (50.0%) | 0/1 (0.0%) | 1/3 (33.3%) |
| *41* | 1/2 (50.0%) | 0/1 (0.0%) | 1/3 (33.3%) |
| Admitted to intensive care during readmission | 1/2 (50.0%) | 0/1 (0.0%) | 1/3 (33.3%) |
| Length of intensive care readmission (days) |  |  |  |
| *25* | 1/1 (100.0%) | - | 1/1 (100.0%) |
| **Blinding** |  |  |  |
| Which allocation does participant think they  received? |  |  |  |
| CCPB | 1/9 (11.1%) | 1/9 (11.1%) | 2/18 (11.1%) |
| HCPB | 2/9 (22.2%) | 0/9 (0.0%) | 2/18 (11.1%) |
| Doesn’t know | 6/9 (66.7%) | 8/9 (88.9%) | 14/18 (77.8%) |
| Of those who named a treatment arm, reason why this answer was given |  |  |  |
| *Question not answered/no reason given* | 2/3 (66.7%) | 0/1 (0.0%) | 2/4 (50.0%) |
| *Felt not completely orientated / alert for 3 days*  *post-surgery* | 0/3 (0.0%) | 1/1 (100.0%) | 1/4 (25.0%) |
| *No postoperative cognitive dysfunction* | 1/3 (33.3%)*^2^* | 0/1 (0.0%) | 1/4 (25.0%) |

*Note: CABG: Coronary Artery Bypass Graft, CCPB: conventional cardiopulmonary bypass, HCPB: hypobaric cardiopulmonary bypass*

*^1^ Follow-up data obtained from hospital records*

*^2^ This participant thought that they were in the HCPB arm*

**Table S7: Baseline demography and baseline quality of life for the AVR + CABG group**

|  | **AVR±CABG** |
| --- | --- |
|  | **(n=6)** |
| **Demography and Quality of Life** | **n/N (%)** |
| **Baseline characteristics** |  |
| Female | 3/6 (50%) |
| Age (mean, SD) | 67.2 (10.5) |
| BMI (mean, SD) | 29.5 (7.1) |
| Ethnicity |  |
| *White or Caucasian* | 6/6 (100%) |
| **Medical history** |  |
| Smoking |  |
| *Yes* | 0/6 (0%) |
| *Ex> 1 month* | 2/6 (33%) |
| *No* | 4/6 (67%) |
| Diabetes |  |
| *Injected medication* | 0/6 (0%) |
| *Oral* | 1/6 (17%) |
| *No* | 5/6 (83%) |
| Hypothyroidism | 0/6 (0%) |
| Cancer | 1/6 (17%) |
| Chronic pulmonary disease | 0/6 (0%) |
| Medically treated hypertension | 4/6 (67%) |
| Medically treated hypercholesterolaemia | 2/6 (33%) |
| Family history (cardiovascular) | 0/6 (0%) |
| Unstable angina | 0/6 (0%) |
| Extracardiac arteriopathy | 0/6 (0%) |
| Myocardial infarction | 4/6 (67%) |
| Stroke | 0/6 (0%) |
| Left ventricular ejection fraction |  |
| *Good (>50%)* | 5/6 (83%) |
| *Moderate (30 – 50%)* | 1/6 (17%) |
| >50% Disease in the left main stem | 0/6 (0%) |
| Heart rhythm |  |
| *Sinus* | 6/6 (100%) |
| Renal impairment |  |
| *Normal (CC>85ml/min)* | 4/6 (67%) |
| *Moderate (CC>85 & CC>50)* | 2/6 (33%) |
| *Severe (CC<50)* | 0/6 (0%) |
| Poor mobility | 0/6 (0%) |
| Previous cardiac surgery | 0/6 (0%) |
| Active endocarditis | 0/6 (0%) |
| Critical preoperative state | 0/6 (0%) |
| NYHA class |  |
| *I* | 2/6 (33%) |
| *II* | 3/6 (50%) |
| *II* | 1/6 (17%) |
| *IV* | 0/6 (0%) |
| CCS class |  |
| *0* | 3/6 (50%) |
| *I* | 0/6 (0%) |
| *II* | 2/6 (33%) |
| *III* | 1/6 (17%) |
| *IV* | 0/6 (0%) |
| Pulmonary hypertension |  |
| *None* | 6/6 (100%) |
| **Operation-related factors** |  |
| Urgency |  |
| *Elective* | 4/6 (67%) |
| *Urgent* | 2/6 (33%) |
| *Emergency* | 0/6 (0%) |
| *Salvage* | 0/6 (0%) |
| Weight of the intervention:- |  |
| *Isolated AVR* | 5/6 (83%) |
| *2 procedures eg. CABG + AVR* | 1/6 (17%) |
| *3 procedures* | 0/6 (0%) |
| Surgery of thoracic aorta | 0/6 (0%) |
| Euroscore II (%, median, IQR) | 1.1 (1.0, 1.4) |
| **Bloods** |  |
| Serum creatinine (µmol/L:median, IQR) | 76 (71, 93) |

*Note: AVR: Aortic Valve Replacement, CABG: Coronary Artery Bypass Graft, CCPB: SD: Standard Deviation, CC: Creatinine clearance, NYHA: New York Heart Association, CCS: Canadian Cardiovascular Society, IQR: Interquartile Range*

**Table S8: Intra-operative and post-operative details for the AVR + CABG group**

|  | **AVR±CABG** |
| --- | --- |
|  | **(n=6)** |
| **Operation details** | **n/N (%)** |
| **General operation details** |  |
| Duration of operation (hours: mean, S.D.) | 3.2 (1.0) |
| Total bypass time (mins: mean, S.D.) | 76 (24) |
| Total cross-clamp time (mins: mean, S.D.) | 58 (16) |
| Type of surgical access  *Full sternotomy* | 6/6 (100.0%) |
| CABG: number of anastomoses |  |
| *2* | 1/1 (100.0%) |
| Type of aortic valve replacement |  |
| *Tissue prosthetic replacement* | 4/6 (66.7%) |
| *Mechanical prosthetic replacement* | 2/6 (33.3%) |
| Lowest core body temperature recorded during  operation (°C; mean, S.D.) | 34.7 (0.5) |
| **Insufflation details** |  |
| Carbon dioxide insufflation used | 0/6 (0.0%) |
| **Myocardial protection** |  |
| Myocardial protection type |  |
| *Blood* | 6/6 (100.0%) |
| *Other* | 0/6 (0.0%) |
| Temperature |  |
| *Warm* | 0/6 (0.0%) |
| *Cooled* | 6/6 (100.0%) |
| Infusion mode |  |
| *Antegrade* | 5/6 (83.3%) |
| *Retrograde and antegrade* | 1/6 (16.7%) |
| Timing |  |
| *Intermittent* | 6/6 (100.0%) |
| **Intra-operative complications** |  |
| Calcified aorta | 0/6 (0.0%) |
| Post-cardiotomy | 0/6 (0.0%) |
| **Post-operative details** |  |
| **General post-operative details** |  |
| Post-operative intensive care stay length (hours: median, IQR) | 9.7 (7.9, 12.2) |
| Post-operative hospital stay length (days: median, IQR) | 7.2 (5.2, 7.9) |
| Clinical evidence of stroke^1^ | 0/6 (0.0%) |
| **Discharge details** |  |
| Discharge destination |  |
| *Hospital discharge home* | 6/6 (100.0%) |
| Discharge delayed | 0/6 (0.0%) |

*Note: AVR: Aortic Valve Replacement, CABG: Coronary Artery Bypass Graft, SD: Standard Deviation, IQR: Interquartile Range*

*^1^ Clinical evidence of cerebral, spinal cord or retinal focal ischemic injury that persisted for more than 24h or until death*

**Table S9: Primary outcome: Trans-cranial doppler ultrasound (TCDU) gaseous microemboli (GME) data for the AVR + CABG group**

|  | **AVR±CABG** |
| --- | --- |
|  | **(n=6)** |
| **Outcome details** | **median, IQR** |
| TCD scan completed (n/N (%)) | 5/6 (83%)^1^ |
| Reason TCD scan not completed (n/N (%)) |  |
| *middle cerebral artery could not be located* | 1/1 (100%) |
| **Right middle cerebral artery**  GME count from:- |  |
| start of CPB to cross-clamp application | 130 (69, 157) |
| cross-clamp application to cross-clamp removal | 215 (160, 247) |
| cross-clamp removal to discontinuation of CPB | 283 (269, 285) |
| discontinuation of CPB to skin closure | 47 (43, 146) |
| Total GME count | 829 (760, 1283) |
| Total GME occurrence rate (counts/min) | 7.1 (6.7, 8.9) |
| **Left middle cerebral artery**  GME count from:- |  |
| start of CPB to cross-clamp application | 126 (6, 184) |
| cross-clamp application to cross-clamp removal | 183 (52, 235) |
| cross-clamp removal to discontinuation of CPB | 210 (181, 221) |
| discontinuation of CPB to skin closure | 30 (20, 142) |
| Total GME count | 668 (437, 747) |
| Total GME occurrence rate (counts/min; median, IQR) | 6.1 (2.6, 6.2) |

*Note: AVR: Aortic Valve Replacement, CABG: Coronary Artery Bypass Graft, TCD: Transcranial Doppler, GME: Gaseous Micro-emboli, CPB: cardiopulmonary bypass, IQR: Interquartile Range.*

*^1^ Count data available for both the right and left middle cerebral artery for all 5 participants*

**Table S10: Other Trans-cranial doppler ultrasound (TCDU) data for the AVR + CABG group**

|  | **AVR±CABG** |
| --- | --- |
|  | **(n=6)** |
| **Outcome details** | **Median, IQR** |
| **PARTICULATE MICROEMBOLI (PME):-** |  |
| **Right middle cerebral artery**^1^  PME count from:- |  |
| start of CPB to cross-clamp application | 40 (23, 49) |
| cross-clamp application to cross-clamp removal | 70 (65, 85) |
| cross-clamp removal to discontinuation of CPB | 106 (104, 109) |
| discontinuation of CPB to skin closure | 27 (16, 67) |
| Total PME count | 390 (259, 507) |
| Total PME occurrence rate (counts/min) | 2.7 (2.4, 4.1) |
| **Left middle cerebral artery**^1^  PME count from:- |  |
| start of CPB to cross-clamp application | 25 (0, 107) |
| cross-clamp application to cross-clamp removal | 32 (24, 47) |
| cross-clamp removal to discontinuation of CPB | 98 (82, 125) |
| discontinuation of CPB to skin closure | 55 (9, 88) |
| Total PME count | 237 (219, 334) |
| Total PME occurrence rate (counts/min) | 1.9 (1.5, 2.0) |
| **ARTEFACTS** |  |
| **Right middle cerebral artery**^1^  Artefact count from:- |  |
| start of CPB to cross-clamp application | 0 (0, 0) |
| cross-clamp application to cross-clamp removal | 0 (0, 1) |
| cross-clamp removal to discontinuation of CPB | 0 (0, 0) |
| discontinuation of CPB to skin closure | 68 (61, 393) |
| Total Artefact count | 70 (61, 393) |
| Total Artefact occurrence rate (counts/min) | 0.5 (0.3, 3.7) |
| **Left middle cerebral artery**^1^  Artefact count from:- |  |
| start of CPB to cross-clamp application | 0 (0, 0) |
| cross-clamp application to cross-clamp removal | 0 (0, 1) |
| cross-clamp removal to discontinuation of CPB | 0 (0, 0) |
| discontinuation of CPB to skin closure | 99 (50, 296) |
| Total Artefact count | 100 (50, 296) |
| Total Artefact occurrence rate (counts/min) | 0.4 (0.4, 2.8) |
| **CLUSTER SHOWER** |  |
| **Right middle cerebral artery**^1^  Cluster shower duration (sec) from:- |  |
| start of CPB to cross-clamp application | 4 (3, 6) |
| cross-clamp application to cross-clamp removal | 40 (26, 65) |
| cross-clamp removal to discontinuation of CPB | 37 (25, 38) |
| discontinuation of CPB to skin closure | 5 (1, 11) |
| **Left middle cerebral artery**^1^  Cluster shower duration (sec) from:- |  |
| start of CPB to cross-clamp application | 4 (0, 7) |
| cross-clamp application to cross-clamp removal | 18 (8, 25) |
| cross-clamp removal to discontinuation of CPB | 22 (16, 36) |
| discontinuation of CPB to skin closure | 4 (1, 20) |
| **TIME-AVERAGED MAXIMUM FLOW VELOCITY** |  |
| **Right middle cerebral artery**^1^  Time-averaged maximum flow velocity (L/min) from:- |  |
| start of CPB to cross-clamp application | 44 (41, 45) |
| cross-clamp application to cross-clamp removal | 47 (42, 49) |
| cross-clamp removal to discontinuation of CPB | 38 (38, 41) |
| discontinuation of CPB to skin closure | 45 (44, 49) |
| **Left middle cerebral artery**^1^  Time-averaged maximum flow velocity (L/min) from:- |  |
| start of CPB to cross-clamp application | 44 (41, 46) |
| cross-clamp application to cross-clamp removal | 49 (48, 51) |
| cross-clamp removal to discontinuation of CPB | 39 (31, 42) |
| discontinuation of CPB to skin closure | 50 (36, 55) |

*Note: : AVR: Aortic Valve Replacement, CABG: Coronary Artery Bypass Graft, IQR: Interquartile Range, PME: Particulate Micro-emboli, CPB: cardiopulmonary bypass.*

*^1^ For all four time periods, data were missing for 1 participant where the middle cerebral artery could not be located.*

**Table S11: Secondary outcome data for the AVR + CABG group**

|  | **AVR±CABG** |
| --- | --- |
|  | **(n=6)** |
| **Outcome details** | **n/N (%)** |
| **Number of CPB circuit gaseous microemboli (from CPB start to 10**  **minutes after CPB end)** |  |
| Size 1 |  |
| 1 | 1/6 (17%) |
| 3 | 2/6 (33%) |
| 6 | 1/6 (17%) |
| 8 | 2/6 (33%) |
| Size 2 |  |
| 0 | 1/6 (17%) |
| 1 | 1/6 (17%) |
| 2 | 1/6 (17%) |
| 3 | 1/6 (17%) |
| 7 | 1/6 (17%) |
| 8 | 1/6 (17%) |
| Size 3 |  |
| 0 | 3/6 (50%) |
| 1 | 1/6 (17%) |
| 6 | 1/6 (17%) |
| 25 | 1/6 (17%) |
| All sizes |  |
| 3 | 2/6 (33%) |
| 5 | 1/6 (17%) |
| 9 | 1/6 (17%) |
| 21 | 1/6 (17%) |
| 41 | 1/6 (17%) |
| **Rate of CPB circuit gaseous microemboli occurrence (from CPB**  **start to 10 minutes after CPB end, counts per hour: median, IQR)** |  |
| Size 1 | 3.6 (1.9, 6.3) |
| Size 2 | 2.6 (1.1, 3.7) |
| Size 3 | 0.5 (0.0, 3.2) |
| All sizes | 7.3 (3.2, 11.1) |
| **Adverse events within 30 days of**  **Surgery** |  |
| Number of in-hospital complications (events/participants) | 29/6 (100%) |
| Number of serious^1^ in-hospital complications (events/participants) | 3/2 (33%) |
| Number of hospital readmissions within 30 days of surgery | 1/6 (17%) |
| Number of SAEs experienced during 30 day follow-up period  (events/participants) | 6/2 (33%) |

*Note: AVR: Aortic Valve Replacement, CABG: Coronary Artery Bypass Graft, CPB: cardiopulmonary bypass, IQR: Interquartile Range, SAE: Serious adverse event.*

*^1^ Serious adverse events are defined as adverse events leading to or prolonging an existing hospitalisation, resulting in persistent or significant disability, leading to congenital anomaly/birth defect, that are life-threatening or cause death, or that constitute another significant medical event*

**Table S12: Detail of postoperative complications for the AVR + CABG group**

|  | **AVR±CABG**  **Overall (n=6)** | |
| --- | --- | --- |
| **Complication type (by MedDRA preferred term)** | **Complications**  **Events/ Participants (%)** | **Serious^1^ complications**  **Events/ Participants (%)** |
| **IN HOSPITAL COMPLICATION** |  |  |
| *Cardiac disorders* |  |  |
| Arrhythmia | 4/4 (67%) | 2/2 (33%) |
| Pericardial effusion | 1/1 (17%) | 0/0 (0%) |
| *Gastrointestinal disorders* |  |  |
| Nausea | 3/3 (50%) | 0/0 (0%) |
| Vomiting | 2/2 (33%) | 0/0 (0%) |
| *General disorders and administration site conditions* |  |  |
| Peripheral oedema | 4/4 (67%) | 0/0 (0%) |
| Post procedural fever | 5/5 (83%) | 0/0 (0%) |
| *Nervous system disorders* |  |  |
| Insomnia | 1/1 (17%) | 0/0 (0%) |
| *Respiratory, thoracic and mediastinal disorders* |  |  |
| Atelectasis | 1/1 (17%) | 0/0 (0%) |
| Pleural effusion | 2/2 (33%) | 1/1 (17%) |
| *Vascular disorders* |  |  |
| Haematoma | 1/1 (17%) | 0/0 (0%) |
| Haemodynamic instability | 5/5 (83%) | 0/0 (0%) |
| *TOTAL IN-HOSPITAL COMPLICATIONS* | *29/6 (100%)* | *3/2 (33%)* |
| **ADVERSE EVENTS FROM DISCHARGE TO 30 DAYS AFTER SURGERY** | |  |
| *General disorders and administration site conditions* |  |  |
| Death | 1/1 (17%) | 1/1 (17%) |
| *Infections and infestations* |  |  |
| Infection | 1/1 (17%) | 1/1 (17%) |
| *Injury, poisoning and procedural complications* |  |  |
| Surgical procedure repeated | 1/1 (17%) | 1/1 (17%) |
| *TOTAL POST-DISCHARGE COMPLICATIONS* | *3/1 (17%)* | *3/1 (17%)* |
| ***OVERALL (TOTAL COMPLICATIONS DURING 30 DAY FOLLOW-UP)*** | 32/6 (100%) | 6/2 (33%) |

*Note: AVR: Aortic Valve Replacement, CABG: Coronary Artery Bypass Graft, MedDRA: Medical Dictionary for Regulatory Activities.*

*^1^ Serious adverse events are defined as adverse events leading to or prolonging an existing hospitalisation, resulting in persistent or significant disability, leading to congenital anomaly/birth defect, that are life-threatening or cause death, or that constitute another significant medical event*
